# How to Develop Reliable Instruments to Measure the Cultural Evolution of Preferences and Feelings in History?

**DOI:** 10.3389/fpsyg.2022.786229

**Published:** 2022-07-18

**Authors:** Mauricio de Jesus Dias Martins, Nicolas Baumard

**Affiliations:** Institut Jean Nicod, Département d'Etudes Cognitives, ENS, EHESS, CNRS, PSL Research University, Paris, France

**Keywords:** NLP, word2vec, factor analyses, historical economics, text analysis, LIWC

## Abstract

While we cannot directly measure the psychological preferences of individuals, and the moral, emotional, and cognitive tendencies of people from the past, we can use cultural artifacts as a window to the zeitgeist of societies in particular historical periods. At present, an increasing number of digitized texts spanning several centuries is available for a computerized analysis. In addition, developments form historical economics have enabled increasingly precise estimations of sociodemographic realities from the past. Crossing these datasets offer a powerful tool to test how the environment changes psychology and *vice versa*. However, designing the appropriate proxies of relevant psychological constructs is not trivial. The gold standard to measure psychological constructs in modern texts – Linguistic Inquiry and Word Count (LIWC) – has been validated by psychometric experimentation with modern participants. However, as a tool to investigate the psychology of the past, the LIWC is limited in two main aspects: (1) it does not cover the entire range of relevant psychological dimensions and (2) the meaning, spelling, and pragmatic use of certain words depend on the historical period from which the fiction work is sampled. These LIWC limitations make the design of custom tools inevitable. However, without psychometric validation, there is uncertainty regarding what exactly is being measured. To overcome these pitfalls, we suggest several internal and external validation procedures, to be conducted prior to diachronic analyses. First, the semantic adequacy of search terms in bags-of-words approaches should be verified by training semantic vector spaces with the historical text corpus using tools like word2vec. Second, we propose factor analyses to evaluate the internal consistency between distinct bag-of-words proxying the same underlying psychological construct. Third, these proxies can be externally validated using prior knowledge on the differences between genres or other literary dimensions. Finally, while LIWC is limited in the analysis of historical documents, it can be used as a sanity check for external validation of custom measures. This procedure allows a robust estimation of psychological constructs and how they change throughout history. Together with historical economics, it also increases our power in testing the relationship between environmental change and the expression of psychological traits from the past.

## Introduction

One of the core missions of social and political sciences is the study of how individual beliefs and values are formed, how they change, and how the interaction between individuals impacts the dynamics of society and of political institutions. Population-wide surveys and opinion polls are one of the main traditional tools to gather information on values and beliefs (European Values Study, 2021; WVS Database, 2021). These data can be used to uncover the relationships between social values, political institutions, group behavior, and the social-economic environment with rigorous quantitative tools (e.g., Hansen et al., 2018; Talavera et al., 2018; Ruck et al., 2019; Knechel and Mintchik, 2020). More recently, an explosion in social media data and the advent of computational social sciences have also allowed a quasi-real-time characterization of beliefs, values, and emotions of social media users, which constitute large segments of the population (Lazer et al., 2009, 2020; Giles, 2012; Mäntylä et al., 2018; Yadav and Vishwakarma, 2020).

While useful, social media and polling data are limited in their temporal scopes. Facebook and Twitter were only founded after 2004, and social media data are not extensive before this period. Moreover, while modern polling of electoral preferences has been conducted in the US since the first half of the 20th century, systematic surveys of values and attitudes are relatively recent (since the 1980's) (European Values Study, 2021; WVS Database, 2021). Thus, different forms of data are required to reconstruct longer time series of relevant psychological and cultural dynamics throughout history.

Fortunately, a second revolution occurred within computational social sciences, particularly in the field of digital humanities, pertaining to the availability of digitized cultural data such as paintings (Morin, 2013; Safra et al., 2020), music (Mehr et al., 2019; Cowen et al., 2020), sculpture (Cowen and Keltner, 2020), political speeches (Barron et al., 2018; Theocharis and Jungherr, 2021), and books, newspapers, and magazines (Acerbi et al., 2013; Iliev et al., 2016; Morin and Acerbi, 2017; Hills et al., 2019; Jackson et al., 2019; Martins and Baumard, 2020). These data, together with the development of new analytical tools, have allowed the generation of meaningful proxies of social preferences and sentiments throughout history. For instance, digital humanities tools have been able to characterize the rise of prosocial preferences prior to democratizing revolutions in the early modern period (Martins and Baumard, 2020), the rise of subjective wellbeing since 1730 till present (Hills et al., 2019), and a decrease in words related to norms of cultural tightness vs. looseness since 1800 (Jackson et al., 2019).

Concomitantly to this revolution in digital humanities, there has been an improvement in the techniques of historical econometrics, improving socioeconomic estimates further into the past. The availability of these time series in systematic datasets (Wrigley et al., 1997; Maddison Project Database, 2018; Clio Infra|Reconstructing Global Inequality, 2019; Our World in Data, 2019) enables the investigation of the relationship between psychological traits and socioeconomic trends. For instance, we can test how living standards in the early modern period (e.g., GDPpc, life expectancy) influence the prevalence of prosocial attitudes in theater plays (Martins and Baumard, 2020) or whether they change art patrons' predispositions to be represented as dominant or trustworthy (Safra et al., 2020). Importantly, we can test not only whether cultural and socioeconomic historical time series correlate but also whether there is evidence of temporal precedence of one over the other. Using cross correlations (Bourke, 1996) and lagged regressions (Cromwell and Terraza, 1994), it is currently possible to inquire whether the expression of certain cultural traits is more likely to precede socioeconomic shifts or *vice versa*.

In sum, developments in computational social sciences, digitized historical culture, and historical econometrics have opened an unprecedented window to quantitatively study the dynamics and determinants of collective behaviors through extended time periods. However, there are some pitfalls in conducting this kind of research, which can undermine the validity of the psychological measures. In this manuscript, we review these pitfalls and suggest a pipeline to overcome some of the challenges faced when conducting historical text analysis.

### The Challenge of Using Text Mining to Explore Historical Sentiments

Within computational social sciences, text analysis is particularly popular. Customizable and sophisticated tools for automated Natural Language Processing (NLP) have been developed for widely used programming languages such as R (Wild, 2021) or Python (Natural Language Toolkit–NLTK 3.6.2 Documentation, 2021). NLP can be used not only to assess the frequency of words, or bags of words, but also to perform more complex sentiment (Liu, 2020) and topic analyses (Řehåřek and Sojka, 2010). Being highly customizable, these tools can be used to describe and detect overall patterns of language use, how they change, and how they are distributed across groups (Serrano-Guerrero et al., 2015; Karjus et al., 2018, 2020; Chen et al., 2021). However, the same techniques are often not suited for psychological research. One of the main challenges is that it is not trivial to validate custom measures, i.e., to ensure that the dimensions measured correspond to actual psychological realities.

The LIWC partially solves this problem and became the gold standard (Pennebaker et al., 2015) in NLP-based psychological research. This tool is connected to a graphic user interface, thus not requiring programming knowledge, and it automatically computes the frequency of dozens of psychologically relevant bags of words such as future orientation, emotions, clout, rationality, and social orientation. The main advantage of LIWC is that its bags of words – and the concepts they represent – have been validated by experimental psychological research. In other words, this tool has been used to analyze written texts of participants who also underwent independent psychometric evaluation, and the bag-of-words frequencies within LIWC have been shown to correlate meaningfully with the relevant psychological dimensions (Pennebaker et al., 2015).

Despite its usefulness to analyze modern texts and social media, LIWC can be limited when used in historical texts. First, historical concepts of interest often fall outside the default set of bags-of-words of LIWC. Second, and more importantly, the dimensions within LIWC have been validated for modern language and with modern participants. The way to express certain ideas and feelings (and even their orthography) changes with time (Karjus et al., 2020) and the bags of words within LIWC may fail to capture the expression of similar concepts in historical texts. For instance, words like “seethe,” “whirlwind,” “spleen,” and “wrath” are commonly used to express Anger in the early modern England but are not included in LIWC bag-of-words for Anger. Thus, when performing analysis of texts from the past, the two main challenges are (1) to flexibly develop tools adapted to the dimensions and time periods that we wish to investigate and (2) to ensure that the extracted measures empirically relate to psychological and social constructs of interest.

In this manuscript, we describe a pipeline with a series of recommendations for researchers doing analysis of texts from the past. First, we recommend using the flexibility of Python/R NLP tools to generate semantic vector spaces, which can then be used to develop historically appropriate bags of words. Then, we review several techniques, which can be used to validate the instrumental measures regarding both their internal coherence and their ecological validity. A practical application of this pipeline can be found in the study by Martins and Baumard (2020) and the scripts for each step in https://osf.io/h5vcq/.

## Materials

First and foremost, conducting research of historical texts requires a quality source dataset. One of the most used datasets for diachronic analysis is google ngram (https://books.google.com/ngrams/info). This dataset is useful for modern analyses, but the coverage decreases as the time window moves further into the past. Another useful source for relatively recent texts, including novels, movie scripts, and spoken dialogue, can be obtained in the dataset CoCa, but these have similar limitations in terms of historical coverage (https://www.english-corpora.org/movies/).

Alternatively, source texts can be obtained from medieval and early modern periods e.g., in English (https://emed.folger.edu/corpus-search, https://www.folgerdigitaltexts.org/download, https://quod.lib.umich.edu/e/eebogroup/, https://quod.lib.umich.edu/e/ecco/), French (http://www.theatre-classique.fr/pages/programmes/PageEdition.php), or Latin (https://comphistsem.org/home.html). Working with the source texts from the past has the advantage that the user can directly verify the preprocessing quality and make necessary adjustments with custom scripts. The procedures and techniques for text preprocessing fall outside of the scope of this manuscript, as they have been extensively covered by other manuals and tutorials and can be performed by standard Python and R toolboxes (Hardeniya et al., 2016; Welbers et al., 2017). Examples of raw and preprocessed texts for the early modern period, and the respective Phyton scripts, can be found in https://osf.io/emxqw/.

The steps enumerated below are implemented with Python toolboxes for NLP and R toolboxes for statistical analysis. Reference to the specific toolboxes and links to example scripts are available in the next section.

## A Roadmap To Investigate Historical Sentiments: Methods And Anticipated Results

**Figure 1:**
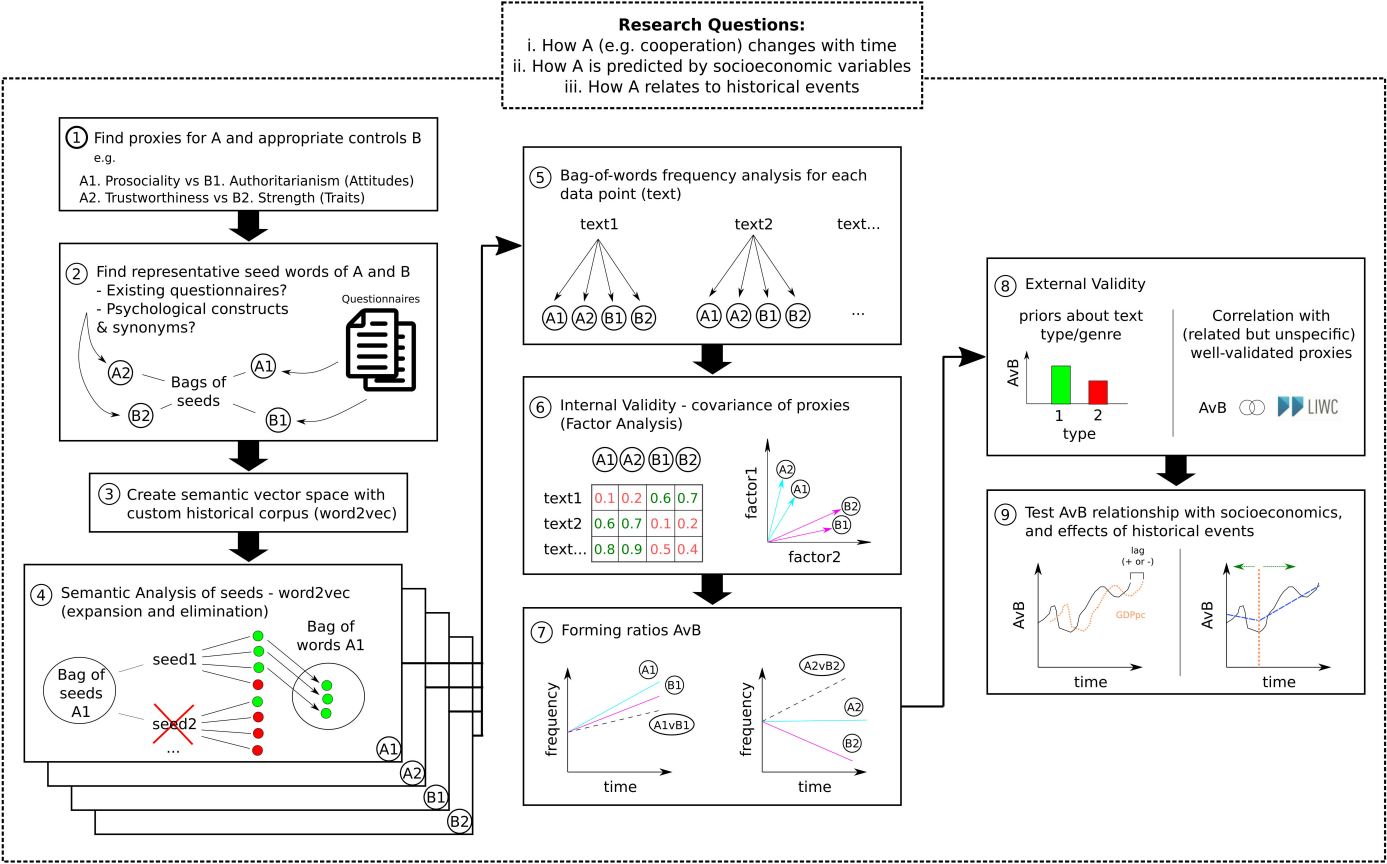
Pipeline for diachronic analysis of historical texts (detailed explanation of each step in the text). **(1) Proxies and controls**. Diachronic analyses require the selection of appropriate proxy measures of target psychological dimensions (A1 and A2) and of control conditions (B1 and B2). For instance, the contrast Cooperation vs. Dominance can be proxied as Prosociality vs. Authoritarianism (Attitudes) or Trustworthiness vs. Strength (Traits). Deriving more than one proxy is crucial for subsequent internal validation (see step 6) and generalisability. **(2) Bags of seeds**. To derive meaningful bags-of-words for each dimension (A1, A2, B1, and B2), it is necessary to find seed words for subsequent exploratory semantic analysis (step 4). A possible approach is to extract central words in existing psychometric questionnaires. For instance, the seed words “Care,” “Support,” and “Assistance” are central in questionnaires measuring individuals' prosociality (Baumsteiger and Siegel, 2019). An alternative or complementary approach is to use dictionary tools such as WordNet (Princeton University, 2010; WordNet Interface, n.d.) to generate a list of synonyms and hyponyms. **(3) Historical semantic map**. The crucial step to generate a historical adequate bags-of-words is to build a semantic vector map of the historical corpus. This enables the exploration of the particular meanings associated with each word in the historical context in step 4. **(4) Bags-of-words**. For each bag-of-seeds (A1, A2, B1, and B2), each seed word is expanded into a set of semantically similar words (within the particular historical context) using word2vec (Mikolov et al., 2013). **(expansion)** The seed word and semantically related terms can be added into a bag-of-words (e.g., “spleen,” “resentment,” “jealousie” are related to “anger” in the early modern period). **(elimination)** The meaning of the seed word can be deemed unspecific and not added to the bags of words (e.g., the word “might” – a synonym of strength–is used more often in the context of “may”/ “should” than of “strength” and should be eliminated). **(5) Frequency analysis**. For each text, compute the total frequency of items in each bag-of-words (A1, A2, B1, and B2). **(6) Internal validity**. To evaluate the coherence between several proxies of the same psychological dimension (A1 and A2) vs. proxies of the control measure (B1 and B2), we can use factor analyses (or other correlation procedures). If the factor analysis does not generate a good separation of the psychological dimensions A and B, it is difficult to determine whether the bag-of-words A1 and A2 are adequate as proxies of A. **(7) Forming ratios AvB**. In diachronic analysis, it is not sufficient to track the dynamics of a psychological variable of interest (A) but rather how it varies in relation to a control variable (B), e.g., using a normalized ratio AvB = (A−B)/(A+B). Using more than one ratio (A1 vs. B1 and A2 vs. B2) can improve generalizability of the results. **(8) External validity**. The final step before diachronic analysis is to check for ecological validity. Does the ratio AvB correlate meaningfully with proxies in NLP tools validated for modern speech (e.g., cooperation and social orientation in LIWC)? Does it correctly distinguish between text genres known to vary in particular dimensions (e.g., tragedies are more violent than comedies)? **(9) Diachronic analysis**. We can test: **(left)** the temporal relationship between the ratio AvB and socioeconomic variables using cross-correlation and lag analyses; **(right)** the influence of historical events in psychology by comparing ratio means (or growth rates) pre and post event.

In this section, we describe a step-by-step pipeline (schematized in Figure 1 and explained in detail later) to investigate (i) how psychological traits, attitudes, and preferences change throughout history, (ii) how they relate to historical events, and (iii) whether they can predict (or be predicted by) socioeconomic trends.

This pipeline assumes that psychological traits, attitudes, and preferences can change over time, both within and between generation. The greater is the precision of historical text dating (in some cases we have the exact year), the greater is the sensitivity to detect this change in smaller temporal scales. Critically, this methodology makes no assumptions as to whether historical events and socioeconomic trends precede a change in the cultural expression of psychological traits, or *vice versa*. The methodologies described in step 9 are designed to detect temporal precedence in both directions (for discussions about the relationship between socioeconomical trends and psychological change, see, e.g., Baumard, 2019; Jackson et al., 2019; Ruck et al., 2019; Martins and Baumard, 2020).

### Step 1. Find Proxies and Appropriate Controls

Words such as “freedom,” “cooperation,” “neurosis,” and their synonyms are not commonly used in historical texts. However, these concepts can be expressed more frequently in other ways. The first challenge is to survey the theoretical and empirical literature for potential proxies of the underlying psychological construct *A*. For instance, prosocial attitudes (*A1*), sympathetic emotions (*A2*), or traits of trustworthiness (*A3*) are associated with cooperative behavior (Oosterhof and Todorov, 2009; Baumsteiger and Siegel, 2019; Cowen and Keltner, 2020; Martins and Baumard, 2020; Safra et al., 2020); thus, bags of words denoting these dimensions could be used as reasonable proxies of cooperation. In addition to defining reasonable proxies, it is essential to define control conditions *B* because variations in target concepts can be confounded by other variables. For instance, if the frequency of words related to happiness increases in a particular period, but so does the frequency of words related to all emotions (e.g., sadness and anger), one cannot conclude that the expression of happiness is meaningfully increasing in that period (especially if, for example, sadness is rising faster). In this example, a ratio of happiness vs. all emotions would be more appropriate. We recommend using a set of different proxies [*A1, A2, A3*, …] and corresponding controls [*B1, B2, B3*, …] in order to (i) allow internal validation of the measurements (see step 6) and (ii) triangulate analysis outputs to increase generalizability.

### Step 2. Generate Bags-of-Seeds

The second step is to generate a set of search terms–seeds–that are representative of the proxies derived in step 1. The most straightforward strategy is to use dictionary tools to obtain synonyms and hyponyms of the proxy base word. For example, using WordNet (Princeton University, 2010; WordNet Interface, n.d.), one can obtain the following synonym/hyponym list of “strength” (e.g., script in https://osf.io/h5vcq/:) [“ruggedness,” “hardiness,” “long-suffering,” “brawniness,” “staying_power,” “heartiness,” “huskiness,” “muscularity,” “dynamism,” “muscle,” “stoutness” “immunity,” “indomitability,” “might,” “brawn,” “robustness,” “stalwartness,” “heftiness,” “capacity,” “soundness,” “power,” “firmness,” “toughness,” “endurance,” “stamina,” “sturdiness,” “vigor,” “validity,” “lustiness,” “tolerance,” “legs,” “good_part,” “sinew,” “vigor,” “sufferance,” “invincibility,” “mightiness,” “long-sufferance,” “invulnerability,” “strength”]. However, for other proxies, it is harder to find meaningful lists of synonyms/hyponyms. For instance, using the same WordNet procedure with the word “authoritarianism” returns the list [“police_state,” “authoritarianism”]. An alternative strategy to derive seed words is to survey the literature for psychometric tools used to evaluate the psychological dimension of interest. In this example, the words “obedience,” “strength,” and “authority” are central in questionnaires assessing authoritarian preferences (Toharudin et al., 2012). Thus, these can be explored as seed words complementing dictionary approaches using WordNet.

### Step 3. Generate Historical Semantic Maps (Word2vec)

The approach above describes the exploration of psychometric tools and dictionaries developed for modern language use. However, our intuitions of language use and word meaning might differ from how language was used in particular historical, regional, or even literary genre contexts. To explore the semantic context in which words are used in the studied corpus, we can use tools like word2vec (Mikolov et al., 2013). The first step is to train a semantic vector space with the historical custom corpus (e.g., script in https://osf.io/h5vcq/).

### Step 4. Generate Bags of Words Using Word2vec

After obtaining a semantic vector space using word2vec, it is possible to assess how a seed word is used in the corpus by evaluating its closest words in the semantic space. For instance, the set of 20 closest words to “obedience” in our corpus are [“obedience,” “duty,” “loyalty,” “gratitude,” “respect,” “reverence,” “affection,” “thankfulness,” “piety,” “observance,” “humility,” “allegiance,” “requital,” “submission,” “fidelity,” “friendship,” “subjection,” “disobedience,” “indulgence,” “precept,” “clemency”]. This set provides a degree of confidence that “obedience” is commonly used with the intended meaning.

In addition to semantic verification, we can also use this process to expand the bag of words in relation to the original seed word. Words like “duty,” “loyalty,” and “reverence” can be reasonably added to the bag-of-words of authoritarianism. We call this process of generating extended bags of words from bags-of-seeds *expansion*. Expansion is also crucial to include words with different orthographies (e.g., jealousie and jealousy), which are captured by this semantic analysis.

Conversely, if the semantic analysis suggests that the meaning of the seed word is somewhat ambiguous or unrelated to the intended meaning, the recommendation is to exclude it from the bag-of-words, a process that we call *elimination*. For instance, the word “brawn” is a synonym of strength (according to WordNet). However, the 20 most similar words in the corpus are [“brawn,” “greasie,” “snout,” “kettle,” “carbonado,” “chine,” “anchovy,” “greasy,” “veal,” “weam,” “cheesecake,” “pullet,” “rump,” “raisin,” “gingerbread,” “pear,” “bum,” “ladle,” “biscuit,” “peahen,” “lobster”]. This suggests that “brawn” is mostly used in the culinary sense in our corpus and including it in the analysis would increase the measurement noise.

### Step 5. Frequency Analysis

In this step, we move the focus of the analysis from the corpus to individual texts. For each text, we compute the total word count per bag-of-words (*A1, A2, A3*, …, *B1, B2, B3*, …) divided by each text total word count.

### Step 6. Internal Validity

To evaluate the coherence between several proxies of the same psychological dimension (*A1, A2, A3*, …) vs. proxies of the control measure (*B1, B2, B3*, …), we can use factor analyses (or other correlation procedures). Factor analysis basically computes the covariance structure of the set of proxies and how they are distributed in a vector space. Spatial proximity in the vector space validates the conceptual proximity between proxies. If proxies purporting to measure different aspects of the same underlying psychological dimension (A1, A2, A3, A4, …) cluster together (vs. proxies of the control measures), this can be taken as supporting evidence that these measures tap into the intended underlying construct.

**Figure 2:**
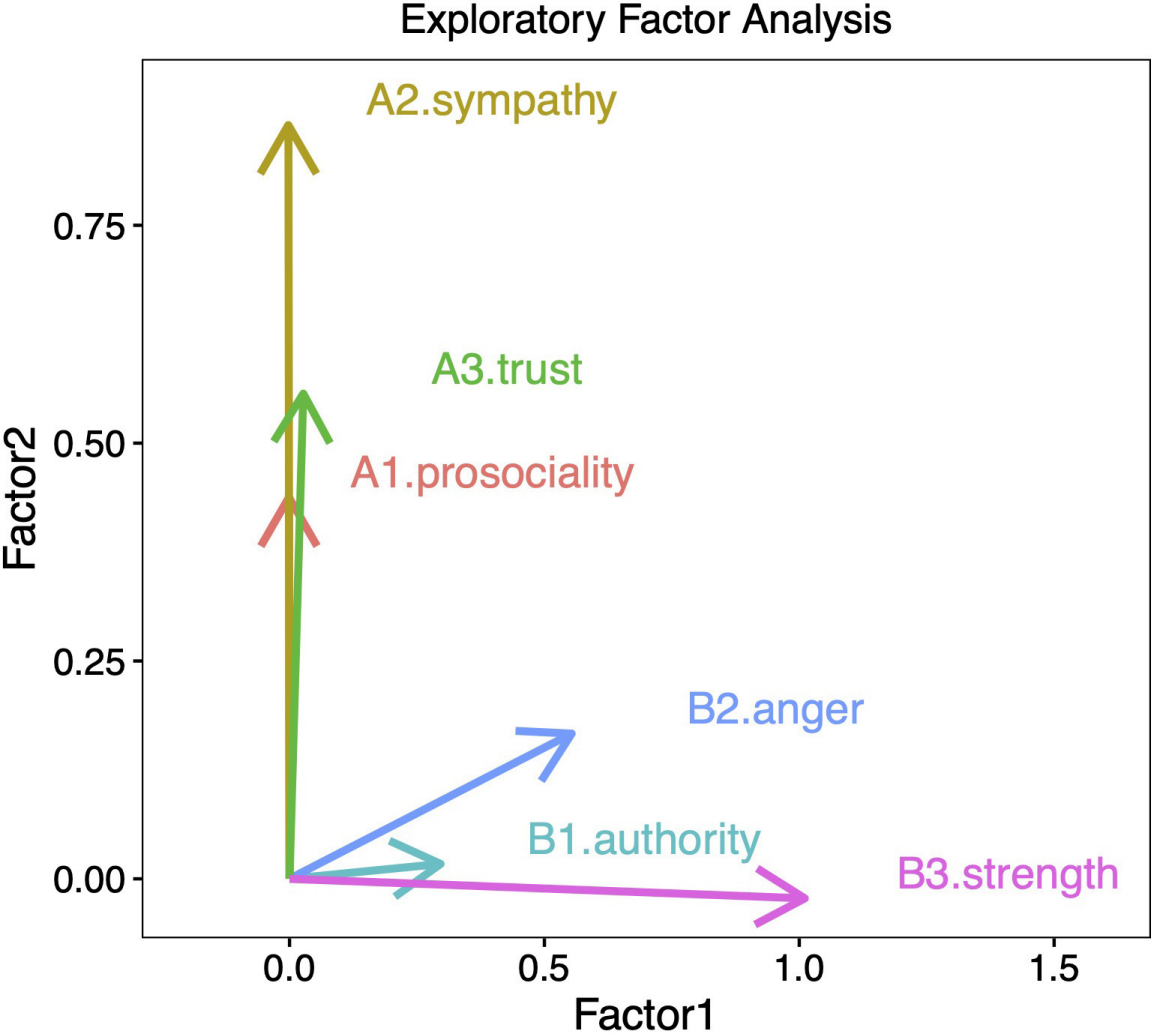
Factor analysis including six variables. Three variables are potentially related to cooperation (prosociality, sympathy, and trustworthiness) and three variables are potentially related to dominance (authority, anger, and strength). The analysis shows that cooperation-related variables load higher on Factor 2, while variables related to dominance load higher in Factor 1 (Martins and Baumard, 2020).

For instance, when investigating the temporal change of cooperation in historical texts, we calculated the frequency of proxies such as prosociality (A1), sympathy (A2), and trustworthiness (A3). Conversely, we computed frequencies of proxies of a control measure of dominance, such as authoritarianism (B1), anger (B2), and strength (B3). Figure 2 illustrates how these bags of words are distributed in a 2D vector space. Visual inspection reveals that A1, A2, and A3 cluster together along the Factor 2 axis, and orthogonally to B1, B2, and B3 (which are higher in Factor 1). This provides supporting evidence that the set of measures A tap into a coherent underlying psychological dimension in contrast to the set of measures B.

Often internal validation is not as successful as in this example, and one or more variables might fall out of their assumed cluster and align more closely with the control than with the target construct. In this case, these variables should not be used. Intuitively relevant bags of words can deviate from the intended underlying meanings for several reasons. For example, words can be used both in affirmative and in negative contexts. If the goal is to determine if characters in fictional stories are kind or unkind, the sentences “John is kind” and “John is not kind” have opposite meanings. Frequency analyses are blind to this distinction, but factor analysis can help determining whether–despite the contextual variation–the bags of words positively tap (on average) into the construct of kindness or its opposite.

In sum, internal validity is crucial to the development of new custom measures to quantify change in psychological traits, attitudes, and preferences. We recommend developing various distinct bags of words tapping into aspects of a target construct (A1, A2, A3, …) and its control (B1, B2, B3, …). In case the exploratory factor analysis fails to detect a clustering of the variance of A1, A2, and A3 on the one hand, and of B1, B2, and B3 on the other hand, there is evidence that the measures are noisy. In this case, we advise going back to the drawing board and developing new bags of words.

### Step 7. Forming Ratios A vs. B

In diachronic analysis, it is not sufficient to track the dynamics of a psychological variable of interest (A) but rather how it varies in relation to a control variable (B). Thus, the operational variable should be a ratio. To avoid outliers and tailed distributions, the ratio can be sensibility calculated to keep the values within a certain range. In our previous research (Martins and Baumard, 2020), for example, we computed a ratio AvB = (A−B)/(A+B), which keeps the values between 1 and −1. To improve generalizability of the results, it is advisable to use more than one ratio (A1 vs. B1, A2 vs. B2, A3 vs. B3, …).

### Step 8. External Validity

**Figure 3:**
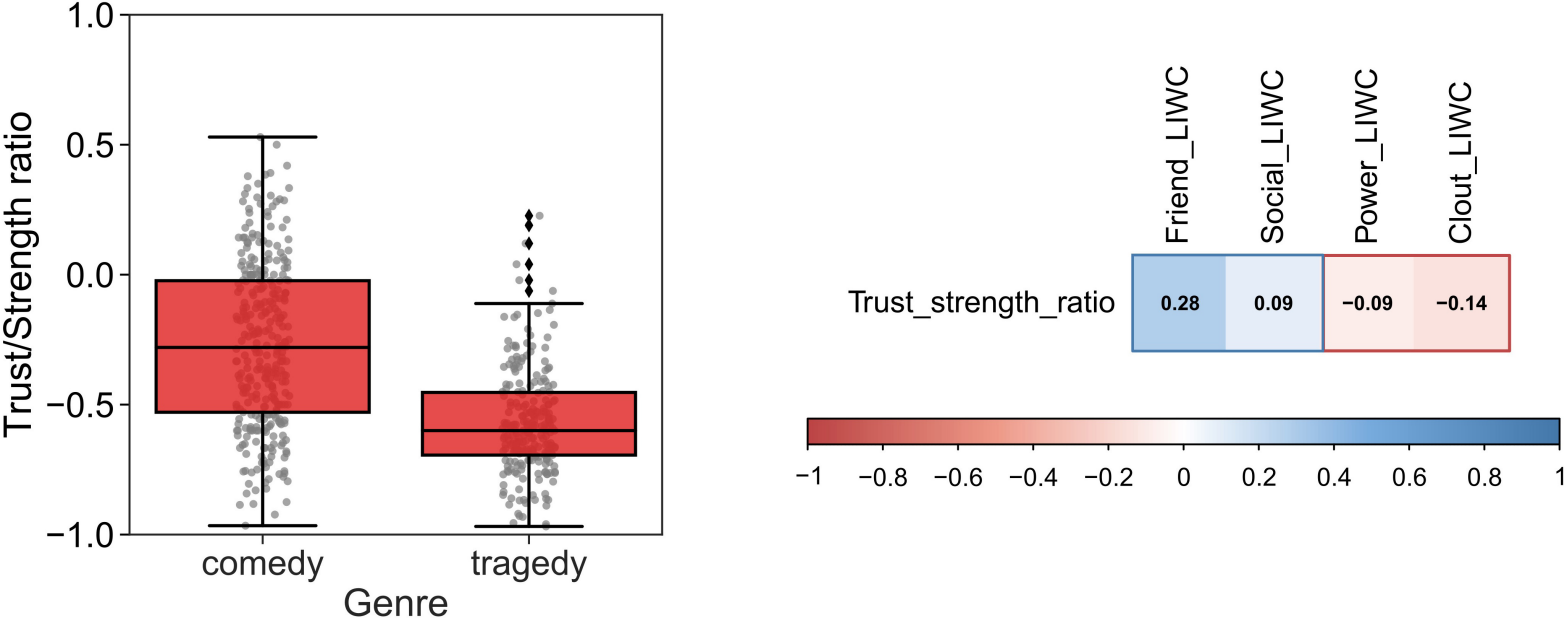
Example of external validation techniques. **(left)** correct distinction between different types of text (the mean ratio Trust/Strength is significantly different between comedies and tragedies, see Martins and Baumard, 2020 for details) **(right)** correlation with indirect proxies from Linguistic Inquiry and Word Count (LIWC).

The final step before diachronic analysis is to check whether the ratios make sensible distinctions between known features of the texts. For instance, it is known that within the genre of theater plays, tragedies are more likely to contain narratives of power than comedies (Nettle, 2005). If a Trustworthiness vs. Strength ratio is higher in comedies than in Tragedies, this provides some ecological validation of the ratio (Figure 3, left). The opposite result would be problematic. In this case, we suggest the rejection of the ratio and going back to the drawing board to explore better alternatives.

Similarly, we can use the dimensions of the LIWC for external validation. LIWC dimensions are limited in scope and psychometrically validated only for modern language users (Pennebaker et al., 2015). However, we can use unspecific LIWC proxies for basic sanity checks. For instance, LIWC does not have specific dimensions for trustworthiness and strength. Nevertheless, the LIWC dimensions of friendship and social orientation can be taken as indirect proxies of trustworthiness; and clout and power can be used as proxies of strength. A positive correlation between Trustworthiness vs. Strength and friendship and social orientation (and negative correlation with clout and power) would provide additional external validation to AvB (Figure 3, right).

Although these correlations can be small (since the LIWC constructs are somewhat unspecific), the general pattern can be indicative of the topic and directionality underling the new construct. This is necessarily an inductive process, which can lend itself to cherry-picking. Hence, we suggest using several different LIWC constructs (e.g., friendship, social orientation, clout, and power) and pre-registering the planned correlation analyses. Finally, both methods of external validation (correlation with LIWC and comparison between categories known to differ *a priori*) should be used.

When the novel measures AvB fail to distinguish between relevant *a priori* categories or display a correlational pattern, which is either nonsignificant or opposite to expected, we suggest going back to the drawing board and developing new bags-of-words.

### Step 9. Diachronic Analysis (Historical and Socioeconomic Relations)

If steps 6 and 8 provide sufficient evidence that the newly developed measures tap into internally coherent and externally valid constructs, it is then safe to proceed to diachronic analyses.

The rapid expansion of datasets of historical socioeconomics (Maddison Project Database, 2018; Clio Infra|Reconstructing Global Inequality, 2019; Our World in Data, 2019) enables the assessment of simple relationships between the psychology of the past and environmental conditions. Mixed models can be used to test how the variation of cultural variables AvB is affected by socioeconomics (e.g., script in https://osf.io/h5vcq/):

AvB = socioeconomic variable 1 + socioeconomic variable 2 + … + year + (1 | author)

Two important controls can be added in this analysis. First is the random effect of author. If certain authors wrote many historical documents, they could excerpt a disproportional influence in the statistics of year-by-year cultural change. In this case, cultural dynamics could not be attributed to historical change, but rather to the idiosyncratic features of one person. Second is the inclusion of “year” as a covariate. Many variables increase linearly with time, thus being well-correlated. However, these secular linear dynamics might not be informative regarding the particular relationship between two variables (e.g., GDPpc and prosociality). By adding “year” as a covariate, we can remove those linear trends and focus on how the temporally local variations of one variable influences the other. In other words, including “year” as a covariate is important to control for other confounding variables, which can affect the secular/long-term trends of both socioeconomical and psychological variables.

To assess the temporal relationship between different variables, we can use time lag analyses (or lag regressions). Time lag analysis is a common tool to assess the causality between two time series X and Y, by determining how well X at time T can be predicted by Y in different points in time, both before and after T (T-n and T+n, respectively) (Cromwell and Terraza, 1994). For example, we can model how AvB at time T – AvB (T) – is predicted by year and 41 additional terms corresponding to the socioeconomic variable with different time lags spanning the interval [T-20, T+20], i.e., ranging from 20 years *before* to 20 years *after* the corresponding time point of AvB (T).

AvB (T) = socioeconomic variable 1 [T-20: T+20] + year + (1 | author)

In this procedure, we first compute the full model containing *all* 41 socioeconomic time lags. Then, we perform model comparison using Bayesian Information Criterion (BIC) and remove socioeconomic lags step-wise until the best model is obtained. Crucially, to prevent overestimation of effects due to temporal autocorrelation, models can be computed using generalized least squares (GLS) (Pinheiro and Bates, 2000) with time (year) as first-order autocorrelation term. The implementation of this procedure in R is available in https://osf.io/h5vcq/.

Finally, diachronic cultural data can also be used to assess how the zeitgeist is affected by particular historical events. When an event is clearly circumspect in time (e.g., wars, revolutions, pandemics, and legislative acts) we can compare the dynamics of AvB before and after the event. In some cases, we are interested in mean differences between periods, for instance, was anxiety more prevalent in cultural expression before or after the terrorist attack of 9/11? In other cases, we are interested in the growth rates of particular variables, for example, was Prosociality vs. Authoritarianism rising faster or slower after a democratic revolution? To compute both the averages and linear trends of each historical period, the model should include the interaction between year and period:

AvB = period (pre-event, post-event) + year + year: period + (1 | author)

The linear trends, or slopes, of each period can be computed with the function emtrends from the package emmeans of R. An implementation can be found in https://osf.io/h5vcq.

## Discussion

Recent advances in digital humanities and historical economics have enhanced the possibility to rigorously test the relationship between culture, psychology, and socioeconomics throughout history. Understanding the processes of social change and mapping their underlying regularities and constraints can be crucial for policy making.

In this manuscript, we briefly reviewed some of the tools and databases, which can be used for this kind of analysis and proposed a pipeline, which avoids the major pitfalls that arise when conducting historical text analysis. This procedure allows a robust estimation of psychological constructs and how they change throughout history. Together with historical economics, it also increases our power in testing the relationship between environmental change and the expression of psychological traits from the past, whichever the directionality.

We successfully used this pipeline in a recent publication in which we demonstrated that the expression of prosocial preferences and values in theater plays were on the rise before the English and French revolutions, and that prosociality in this period was predicted by living standards (Martins and Baumard, 2020). In addition, we applied the same procedure to investigate the evolution of romantic love and of numeric cognition in the early modern period with promising results (https://osf.io/b9msn/, https://osf.io/ka3th/).

To facilitate access to our tools and techniques for early users of natural language processing, we made our pipeline explicit and uploaded example scripts and datasets. With this contribution, we hope to facilitate the extension of this kind of research to other questions concerning the relationship between socioeconomics, history, culture, and psychology.

## Data Availability Statement

Publicly available datasets were analyzed in this study. This data can be found here: https://osf.io/h5vcq.

## Author Contributions

MM and NB contributed to writing the manuscript. MM wrote the python scripts. Both authors contributed to the article and approved the submitted version.

## Funding

This study was funded by the grants EUR FrontCog ANR-17-EURE-0017 and ANR-10-IDEX-0001-02 to PSL, and the grant ANR-19-CE38-0002 to NB.

## Conflict of Interest

The authors declare that the research was conducted in the absence of any commercial or financial relationships that could be construed as a potential conflict of interest.

## Publisher's Note

All claims expressed in this article are solely those of the authors and do not necessarily represent those of their affiliated organizations, or those of the publisher, the editors and the reviewers. Any product that may be evaluated in this article, or claim that may be made by its manufacturer, is not guaranteed or endorsed by the publisher.
